# Rethinking Sexual Citizenship

**DOI:** 10.1177/0038038515609024

**Published:** 2016-09-30

**Authors:** Diane Richardson

**Affiliations:** Newcastle University, UK

**Keywords:** heteronormativity, homonationalism, intimate citizenship, LGBT politics, orientalism, sexual citizenship

## Abstract

Over the last two decades sexuality has emerged as a key theme in debates about citizenship, leading to the development of the concept of sexual citizenship. This article reviews this literature and identifies four main areas of critical framing: work that contests the significance of sexuality to citizenship; critiques that focus on the possibilities and limitations of mobilising the language of citizenship in sexual politics; analyses of sexual citizenship in relation to nationalisms and border making; and literature that critically examines western constructions of sexuality and sexual politics underpinning understandings of sexual citizenship. In order to progress the field theoretically, the article seeks to extend critiques of sexual citizenship focusing on two key aspects of its construction: the sexual citizen-subject and spaces of sexual citizenship. It argues for a critical rethink that encompasses a de-centring of a ‘western-centric’ focus in order to advance understandings of how sexual citizenship operates both in the Global North and South.

## Introduction

In the early 1990s a particular kind of sexual politics emerged that, along with other new social movements, sought to articulate struggles for equality and social justice in terms of the language of citizenship at both national and international levels (D’ Emilio, 2000; Tremblay et al., 2011). These developments, alongside the wider attention given to the concept of citizenship more generally within social and political theory, prompted a new literature that brought discourses of sexuality together with discourses of citizenship. More than two decades later this literature has rapidly expanded to become an important area of study across a number of disciplines, encompassing a complex set of debates over the impact of contemporary sexual politics on the reconfiguration of citizenship including: the transformative power of civic inclusion to change meanings of citizenship *and* sexuality both at the level of social institutions such as marriage and family (e.g. Barker, 2013; Calhoun, 2000; Heaphy et al., 2013; Stacey, 2012; Weeks et al., 2001) and at the level of individual subjectivities (e.g. Bech, 1997; Richardson, 2004; Seidman, 2004); the potential exclusionary effects of processes of ‘sexual democratisation’ (e.g. Butler, 2004; Warner, 1999); questions of nationalism and national border making connected with ideas about modernity and tolerance (e.g. Ammaturo, 2015; Bhattacharyya, 2008; Kahlina, 2015; Mepschen et al., 2010; Puar, 2007); the relationship between forms of neoliberal governance and the politics of sexuality (e.g. Duggan, 2002, 2003; Richardson, 2005, 2015); processes of commodification and consumerism (e.g. Bell and Binnie, 2000; Evans, 1993); and the ways in which constructions of sexual citizenship constitute neo-orientalist and colonial practices (e.g. Altman, 2001; Binnie, 2004; El-Tayeb, 2011; Massad, 2007; Sabsay, 2012). It is therefore prescient to take stock of this burgeoning field both to understand how the concept of sexual citizenship has developed and, more importantly, to assess critically the implications of this legacy for future conceptual and empirical development.

This is the focus of this article, which examines the main themes and epistemological frameworks that are associated with scholarship on sexual citizenship and makes two key contributions. First, it provides a timely and strategic review of the literature published since the emergence of the concept of sexual citizenship in the early 1990s through to 2015. Material was collected through internet and library searches, and snowballing from identified sources; and then analysed thematically. On the basis of the analysis, I argue that this body of work can be grouped into four distinguishable strands of critical debate: work that contests the significance of sexuality to citizenship; critiques that focus on the possibilities and limitations of mobilising the language of citizenship in sexual politics; analyses of sexual citizenship in relation to nationalisms and border making; and literature that critically examines western constructions of sexuality and sexual democracy underpinning understandings of sexual citizenship. The review also demonstrated how the focus of scholarly interest has shifted over the years. For instance, much of the early writing on sexuality and citizenship was conceptual and developed theoretical insights that fed into debates in citizenship studies and social and political theory more broadly. In recent years, however, the sexual citizenship literature has increasingly focused on analysing rights claims such as, for example, the right to equal marriage, prompted by legislative and social changes that have led to new forms of citizenship status for lesbian, gay, bisexual, transgender (LGBT) people in many parts of the world. While recognising the importance of such analyses, this article argues that there is a need for a renewed focus on theoretical approaches to sexuality and citizenship.

From the analysis of the literature it was clear that those writers who *have* advanced theoretical critiques have in the main tended to focus on the ‘sexual’ in sexual citizenship, analysing both the significance of sexuality to citizenship as well as the consequences of particularistic assumptions about sexuality and sexual politics that are ‘western-centric’. This article extends these critiques in a substantially different way by interrogating the normative underpinnings of the concept of sexual citizenship that extend beyond assumptions about sexuality and sexual politics, focusing on the conceptual limitations of a Euro-North American historical configuration. Analyses of how concepts are shaped by the contexts in which they emerge, the ways in which they ‘travel’ across borders, how they are received and re-articulated in new contexts and the possible consequences of this, is a significant feature of postcolonial and feminist writing (Connell, 2007; Spivak, 1988). Here, the question being asked is whether as it is currently imagined the concept of sexual citizenship ‘travels’ well? This is a question that has received little attention in the literature. Indeed, it would be an understatement to claim that there has been little critique of the normative underpinnings of sexual citizenship. This article addresses this gap and advances theorisations of sexual citizenship by focusing on two key aspects of its conceptual construction: the sexual citizen-subject and spaces of sexual citizenship.

Before going on to develop the arguments advanced in this article, it is important to clarify use of terms. Scholarship in the field of gender and sexuality studies has developed substantially over the last 25 years, highlighting the complexities of meaning associated with the terms sexuality and gender. In this article sexuality and gender are seen as socially constructed phenomena that include gendered and sexual categories and associated identities such as lesbian, gay, bisexual, transgender, queer, as well as the acronym LGBT.

## Defining Sexual Citizenship

The work of sociologists beginning with Evans (1993) and followed by the work of writers such as Richardson (1998), Plummer (2003) and Weeks (1998) was key to the emergence of a literature on sexuality and citizenship. Work in other disciplines also contributed to the development of this ‘new’ area of study including in human geography (Bell and Binnie, 2000), political science (Phelan, 1995, 2001; Wilson, 2009) and literary criticism (Berlant, 1997) though, with some notable exceptions (e.g. Cooper, 1995; Cossman, 2007; Robson and Kessler, 2008; Stychin, 2003), its development has remained mainly outside of law. It would not be entirely correct, however, to assume that this literature had no antecedents; feminist scholarship in particular has highlighted issues that we might nowadays refer to by ‘introducing notions of “sexual citizenship” and “intimate citizenship”’ (Segal, 2013: 78). In the 1980s, for example, Pateman (1988) argued that ‘the sexual contract’ is fundamental to ideas of citizenship, highlighting the significance of a married heterosexual context as the norm for full citizen status. In her analysis, however, Pateman does not point out that this involves the privileging of heterosexuality as much as the privileging of a particular form of masculinity (Johnson, 2002). This, in part, may reflect a tendency in much feminist theory to assume that gender and sexuality have to be examined together, with gender typically taking precedence over sexuality (Richardson, 2007).

The meaning of the term sexual citizenship is not self-evident. Sexual citizenship is a multi-faceted concept, understood in a variety of different ways. Some writers, for example, prefer to use the term ‘intimate citizenship’ on the grounds that this allows consideration of a broader range of areas of ‘intimate and personal life’ than what are typically designated as ‘sexual’ (Olesky, 2009; Plummer, 1995, 2003). Surveying this literature it is, however, possible to identify a number of distinct strands of work. First, some analyses focus on sexual citizenship as a way of theorising access to rights granted or denied to different social groups on the basis of sexuality, including but not restricted to rights of sexual expression and identity (Kaplan, 1997; Richardson, 2000).^1^ A great deal of what has been written about sexual citizenship in relation to rights has been in the context of transformations in citizenship status that have been happening in western neoliberal democracies, although recent work is beginning to address this and look beyond ‘the West’ (Lennox and Waites, 2013). The majority of this literature focuses on the rights of non-heterosexual and non-gender normative people through analysis of, in the main, lesbian and gay citizenship, but also incorporating work on bisexual citizenship (Evans, 1993; Monro, 2015; Richardson and Monro, 2012), trans citizenship (Hines, 2009; Monro, 2005; Whittle, 2002) and intersex citizenship (Grabham, 2007), prompted by the recognition of new forms of citizenship status among lesbian, gay, bisexual, transgender and intersexed (LGBTI) populations in many countries in response to various rights demands (Bamforth, 2012; Cossman, 2007). In recent years there has also been work on the status of sexual citizenship in relation to specific issues including, for example, disability (Shildrick, 2013; Siebers, 2008), lesbian motherhood (Ryan-Flood, 2009) and the rights of sex workers (Sabsay, 2013).

Another strand of work conceives of sexual citizenship as primarily about rights to participation in consumer society, linked to marketisation and the consumption of goods and services. In one of the early works on sexual citizenship Evans (1993), for example, argued that sexual citizenship is materially constructed through the dynamics of late capitalism, in particular through practices of consumption. He states that: ‘[s]exual citizenship involves partial, private and primarily leisure and lifestyle membership’, where sexual citizenship rights are chiefly expressed through ‘participation in commercial “private” territories’ (Evans, 1993: 63–64). Bell and Binnie (2000: 96) make a similar argument, claiming that ‘the power that queer citizens enjoy is largely dependent on access to capital and credit’. More recent research has also highlighted the materiality of sexual citizenship in a slightly different sense through a consideration of the significance of class to the forms of sexual citizenship made possible through modes of state recognition such as civil recognition of domestic partnerships, including the right to marry (Taylor, 2011).

A great deal of the literature, however, has been concerned with analysing the underlying assumptions embedded in frameworks of citizenship and the practice of policy to demonstrate how, in addition to being informed by ideas about gender, race and class, understandings of citizenship are historically grounded in normative assumptions about sexuality (Bell and Binnie, 2000; Canaday, 2011; Phelan, 2001; Richardson, 1998; Weeks, 1998). Traditional concepts of citizenship in this sense can be understood as referring to a specific form of sexual citizenship, defined in terms of hegemonic married heterosexual practices. These analyses of sexual citizenship can then be read as a queering of citizenship, opening up the possibility of transforming the norms of citizenship as a whole.

However, while the terms of citizenship may have been queried, it is important to recognise the extent to which there are continuities between definitions of citizenship more generally and notions of sexual citizenship. Although sexual citizenship has brought into focus issues that had previously been taken for granted or ignored in accounts of citizenship concerning bodies, identities and relationships, it nevertheless retains and leaves unquestioned many conventional features of liberal western frameworks of citizenship (see also Sabsay, 2012).

This was apparent in the review of literature on sexuality and citizenship, which reflects many of the issues that cut across citizenship studies more broadly including: understandings of the public and the private, universalism and differentiation; processes of normalisation and the production of new ‘others’; the ways in which majorities as well as minorities construct themselves in relation to one another, as well as what is involved in being recognised as a member of a community and the disciplinary requirements of citizenship. In addition, analyses that focus on sexual citizenship as a way of theorising exclusions from various forms of rights of citizenship based on sexuality mirror the long-standing connections which recognise marriage, parenthood and military service as the key foundations of the liberal regime of modern citizenship (Pateman, 1988). As a consequence, although debates about sexual citizenship may have led to different imaginings of citizenship that encompass ‘private’ practices and highlight the sexualised norms through which citizenship is constituted, a number of general understandings have been carried over from liberal concepts of citizenship to sexual citizenship (see also Bamforth, 2012). I will return to this in the final part of the article. Having examined how different writers have analysed the concept of sexual citizenship, the following section identifies four critical framings within the literature.

## Critical Framings

### The Significance of Sexuality?

As outlined above, one of the main strands of argument in the sexual citizenship literature is that hegemonic forms of heterosexuality underpin constructions of citizenship. Some writers, however, contest the significance attributed to sexuality arguing instead for the centrality of reproduction to contemporary models of citizenship, especially in western societies with modest rates of reproduction. In his elaboration of this argument, Turner (2008: 53) defines the concept of ‘reproductive citizenship’ as concerned with issues of ‘with whom one may reproduce and under what social and legal conditions’, and involving ‘primarily the rights and duties of parenting’. The connection between sexuality and citizenship, Turner argues, is subordinate to the connection between citizenship and reproduction. From this perspective it is not sexuality per se that is understood to be the reason for the historical exclusion of lesbians and gay men from full citizenship, but the non-reproductivity of same sex unions and, therefore, their failure to contribute fully to society. While recognising the significance of reproduction to contemporary citizenship, various writers have responded to such critiques by arguing for a more careful delineation of the relationship between sexuality, reproduction and citizenship than Turner offers, highlighting how the extension of parenting rights remains one of the most contentious areas of LGBT inclusion either through adoption or access to assisted reproductive technology (Eggert and Engeli, 2015; Payne, 2013; Ryan-Flood, 2009). The question that arises is ‘whether individuals become “good citizens” simply by fulfilling their obligations to society in being (re)productive and shouldering the burden of raising children, or whether this is influenced by how they become parents and in what relational contexts?’ (Richardson and Monro, 2012: 64). In other words, it is important to distinguish the mode of reproduction where ‘reproductive heterosex’, specifically in marital relationships and via ‘natural’ conception, remains the norm for good reproductive citizenship (Riggs and Due, 2013).

A different kind of questioning of the significance of sexuality to citizenship is associated with the moves towards cultural normalisation and social inclusion of lesbians and gay men in the UK and in many European countries, as well as elsewhere in the world, including in Canada, Australia and parts of the USA. This has led some writers to ask whether arguments over the (hetero)sexualisation of citizenship need to be revised in light of social and legislative change advancing LGBT equalities. As Seidman (2009) also argues, it seems clear that there has been some loosening of the status of heterosexuality as a condition of institutional belonging. What is more contested, however, is how far the expansion of spaces of citizenship to lesbians and gay men represents a challenge to heteronormative assumptions or, in fact, ‘upholds and sustains them’ (Duggan, 2002: 179).

### Costs of Recognition

In addition to studies of how exclusion from full citizenship has been challenged by lesbian and gay social movements, and the ways in which nation states are responding to LGBT recognition claims, some writers have focused more critically on the costs of recognition, examining new or altered forms of conditionality associated with access to rights of citizenship and the potential exclusions that normalisation as ‘ordinary citizens’ engenders (Duggan, 2002; Warner, 1999). They ask how these moments of sexual democratisation can serve as a means for establishing new boundaries in relation to sexuality that are constitutive of new exclusions and new ‘others’, as well as new belongings. A key theme in this strand of work is the extent to which these new moments of citizenship can be understood as a process of assimilation into the mainstream, upholding normative frameworks for social inclusion *or* as having transformative potential (Barker, 2013). This aspect of the contemporary sexual citizenship agenda has been subject to considerable critical debate, with some writers arguing that legislative and policy change can be transformative including, for example, analyses of how social institutions such as marriage and family might be altered as a consequence of civil recognition of lesbian and gay domestic partnerships and access to parenting rights (Calhoun, 2000; Heaphy et al., 2013; Stacey, 2012), and of the possibilities of changing individual subjectivities – what it might mean to identify as lesbian or gay. One argument is that this ‘conditionality’ demands a particular modality of sexual citizenship, one that is privatised, ‘de-politicised’, ‘de-eroticised’ and domesticated (Warner, 1999) and likely to lead to a de-centring of sexual identity (Bech, 1997; Seidman, 2004). Duggan (2002) refers to this as the ‘new homonormativity’, a concept that has proven to be very influential in the literature. Far less explored in the literature, however, is how extending forms of citizenship to LGBT populations might reconfigure heterosexual subjectivities (Dean, 2014; Richardson, 2004).

This body of work incorporates broader critiques of the ‘turn to citizenship’ in sexual politics that assert that one of the costs of recognition, in emphasising individual rather than collective rights, is that the operations of power and the role of social institutions that sustain gendered and sexualised inequalities are disguised, which makes addressing them more difficult (Richardson and Monro, 2012). A particular focus is on the impact of neoliberalism on LGBT social movements that use neoliberalised forms of citizenship emphasising consumption, rights and ‘family values’ to frame their arguments, and the possible consequential effects of this on activism (Duggan, 2003; Paternotte and Tremblay, 2015).

### Nationalisms and Border Making

A third area of debate within the sexual citizenship literature is associated with analyses of the relationship between sexuality and nationalisms, where a key focus is on how struggles for sexual citizenship have come to act as a performative of the nation-state; a symbolic marker of in/tolerant countries and constructions of ‘modernity’/‘backwardness’. Various writers have examined these processes of national boundary making, highlighting how definitions of western late modernity are constructed through racialised accounts of ‘sexual democracy’ in ways that can be used to justify neo-orientalist and colonial practices (Sabsay, 2012). Puar’s work has been particularly influential in this respect in arguing that in the US context there has been a displacement of heteronormative nationalism and the emergence of new homonormative forms of nationalism associated with cultural othering and border making (Puar, 2007; Puar and Rai, 2002). She uses the term ‘homonationalism’ to describe the way in which lesbian and gay politics have been implicated in national imaginaries in the USA during the ‘war on terror’, linking this with Islamophobic discourses that present Muslim cultures as ‘sexually backward’ as well as oppressive to women (see also Bhattacharyya, 2008; Haritaworn et al., 2008).

In a similar vein, various writers argue that the deployment of sexualities equalities initiatives signifies European identity as ‘cosmopolitan’ associated with liberal progress and tolerance which can serve racist purposes (El-Tayeb, 2011; Mepschen et al., 2010). This joining of homophobic and nationalist discourses has been analysed in terms of the ‘Europeanisation’ of sexual citizenship, where the rights of sexual minorities are now part of EU conditionality, highlighting how parts of Eastern Europe in particular are constructed as ‘backward’ and not ‘civilised’ enough (Ammaturo, 2015; Kahlina, 2015; Stychin, 2003).

### Locating Sexual Citizenship

In addition to analyses of the ways in which nation-states are configured, interest in examining how debates about sexual citizenship may (re)articulate forms of western imperialism includes work that examines the locations of concepts and theories, as well as forms of activism. A particular focus is on the extension of western framings of sexual categories to non-western contexts at the risk of ignoring local understandings and cultural meanings about sexualities. This is a process that Browne et al. (2010) describe in relation to sexualities in the Global South^2^ as colonial regimes of knowledge constituting neo-colonial practices. Puar (2007) has addressed these issues also, arguing for the need to be attentive to what circulates as ‘global’ definitions of lesbian and gay identities and politics (see also Altman, 2001; Massad, 2007).

Critiques of sexual citizenship that have focused on discourses about *citizenship*, as distinct from understandings of sexuality, include postcolonial, queer (of colour especially) and feminist analyses of the constructions of citizenship underpinning arguments for the rights of sexual and gender minorities. The influence of political agendas and strategies of international lesbian and gay movements, often defined as global though originating in the West, has received critical attention within this body of work. Various writers have highlighted how the terms of western lesbian and gay politics can delimit and/or obscure political activism in relation to other cultural situations via misleading ‘universalising’ approaches connected with the ‘human rights turn’ in lesbian and gay movements (Waites, 2009) and the scaling up and professionalisation of lesbian and gay politics that has occurred over the last two decades (Richardson, 2005). Kollman and Waites (2009: 6) argue that this has contributed to ‘making the human rights framing of LGBT politics increasingly dominant in numerous national settings’. This has prompted debate about not only the problems of advocating and organising from a concept of human rights rather than say concepts such as social justice or queer politics (see Duggan, 2003), but also the broader implications of the establishment of so-called global norms of lesbian and gay rights demands. The charge is that it is primarily definitions from the USA and Europe that have colonised ideas of the ‘universal’ in relation to sexual and gender ‘minorities’, resulting in a ‘westernisation’ of LGBT identities *and* politics (Altman, 2001; Binnie, 2004). Long (2009), for example, examines this in relation to the situation in Iran. Massad (2007), in his discussion of the representation of sexualities in the Arab world, similarly argues that western values and assumptions underpin contemporary understandings of ‘sexual democracy’, identifying this as a new form of sexual imperialism based on a universalist ‘sexual epistemology’. However, as these writers are also aware, in highlighting the risk that local meanings and practices may be undermined by colonising processes it is important to be careful not to ignore the complexity of interactions between local sites and global contexts, which can be productive of new ‘hybrid’ identities and political goals (Cruz-Malavé and Manalansan, 2002).

In addition to analyses of the ways in which new configurations of sexuality and its relation to citizenship intersect with race, ethnicity and nationalism to (re)produce neo-orientalist and colonial practices, a few writers have focused on processes of cultural othering and border making at a different scale through critiques of the concept of sexual citizenship itself (Plummer, 2003; Richardson and Monro, 2012; Sabsay, 2012). This is the focus of the second part of the article, which extends this fourth area of critical framing of sexual citizenship by addressing the limits to, and conceptual limitations of, a concept that has largely been configured in Euro-North American terms. Here the aim is not merely to problematise the concept of sexual citizenship in order to produce a more inclusive theoretical account, it is also to draw attention to the normative and normalising effects of its use as currently imagined both within the academy and beyond. In other words, how does the conceptual framing of sexual citizenship border understandings in ways that are constitutive of processes of exclusion and cultural othering? This critique is developed through an examination of two distinct aspects of the epistemological framing of sexual citizenship: the sexual citizen-subject and spaces of sexual citizenship.

## Rethinking Sexual Citizenship

### Constructing Sexual Citizens

What versions of the sexual citizen are constructed in the literature, and how does this condition who can be read as the sexual subject of rights? Analysis of the terms through which discussion of sexual citizenship is typically articulated highlights the mobilisation of a particular kind of self, a particular kind of subject as entitled to claim rights. The liberal notion of an atomistic autonomous subject who can exercise a range of individual choices – in this case in relation to intimacies, sexual identities and relations – is central to debates about sexual and intimate citizenship. The concept of individual choice is a key aspect of western models of neoliberal citizenship and we see this manifested in ideas of sexual and intimate citizenship, where there is a predominant emphasis on the *right to choose* – your partner; whether to marry or not; to have a child or not; your sexual activities (Plummer, 2003). This is a discourse of individual entitlement: the focus is on the individual ‘choosing’ subject (albeit as good neoliberal responsibilised consuming citizens). Constitutional litigation in the USA concerning equal marriage, for example, provides a useful illustration of this, where the centrality of choice to marriage has been an important part of the argument that it is illegitimate to exclude same sex couples (see Bamforth, 2012).

I do not intend to engage here with broader debates over liberalism and concepts of choice (for a useful discussion in relation to sexuality and gender see Chambers, 2007). Rather, in drawing on these debates, this article seeks to highlight (at least) four problems with this conceptual framing of sexual citizenship. First, the privileging of individual rights, rooted in an ideology of individualism, is problematic in societies where constructions of selfhood are experienced differently, as constituted within the social relations of kinship, family and community. Prins (2006), for example, discusses this in relation to how Muslim feminists express a different view of feminist citizenship than their liberal counterparts, employing a more communitarian conception of the individual as an ‘embedded self’. Similarly Joseph’s (1997) analysis of citizenship in Lebanon stresses relationality as a central aspect of selfhood, which affects how rights are experienced as emerging from embedded relationships rather than as ‘belonging’ to atomised individual citizens.

Second, the focus on the individual rights bearing subject is also problematic in cultural contexts where the primary focus is on collective rights. One of the main ways in which sexual citizenship has so far been addressed in the literature is in the context of analysing the effects of the dominant trend in political discourse of LGBT organisations in North America, Australia, New Zealand and Europe towards defining the goals and strategies associated with activism in terms both of universal human rights rhetoric and in demanding rights of citizenship pertaining to being equal ‘ordinary citizens’ of a particular nation-state. In both cases this represents a focus on the rights of individuals rather than group rights such as, for example, demands for ‘gay rights’. Here, it is important to acknowledge situations where the focus is instead on the rights of social groups, collective rights, as distinct from the individualistic assumptions embedded in western models of liberal sexual/citizenship (Brown, 2006; Phillips, 2006). Third, what is also at issue here is the relationship between the state and the subject/citizen. In some countries rights are not conferred through the state but are governed through a person’s relationship with her or his local community or through kin relationships (Richardson et al., forthcoming).

Lastly, the notion of the choosing sexual citizen raises questions over the limits to choice in relation to ‘sexual democracy’. Plummer (2005: 93, emphases in original), for example, argues that the concept of intimate citizenship (his preferred term) demands further clarification ‘for people who often have *little control over* their bodies, feelings, relationships; … and *few socially grounded choices about* identities, gender experiences, erotic experiences’. In a literature where choice narratives dominate, it is important to recognise that the legibility of choice is contingent on social, economic and cultural capital. The possibilities of choosing who to live with, for example, are mediated by the extent to which there is a meaningful context of choice, not only socially and economically but also in terms of culturally conditioned personal imaginaries of choice. In many societies the conception of the individuated world of choice is far removed from everyday reality and certain forms of sexual citizenship may not be intelligible as choices. For example, this construction of the sexual citizen hardly addresses cultural traditions where partner choice may be embedded in family and kinship arrangements limiting opportunities for more individualised choices in sexual relationships and intimate partnerships. In a similar vein, capacity to exercise choice is likely to be restricted in societies where typically women’s economic dependence on men is a primary source of livelihood, there is near universality of heterosexual marriage and there are significant social, economic and personal costs to contesting (hetero)normative assumptions about sexuality and gender. Kabeer (2012), for example, offers a useful illustration of this in her discussion of women and citizenship in Afghanistan and Bangladesh (see also Richardson et al., 2009 on women’s citizenship in Nepal). We need, therefore, to acknowledge the socially and culturally constructed spaces for individual autonomy and decision making in relation to different forms of sexual citizenship. In this way, ‘individualised’ choice must always be considered alongside intersecting forms of social inequality.

How then might we construe sexual citizenship if we decouple it from individuation and the choosing citizen-subject? There is a need to develop conceptual understandings beyond what has so far been an emphasis upon a ‘politics of choice’, detailing what both enables and constrains how people experience sexual and intimate lives across different societies and different groups within these.

## Spaces of Sexual Citizenship

This section examines the loci of sexual citizenship, and highlights the centrality of liberal notions of privacy within the sexual citizenship literature, both in terms of the private sphere as a space to make claims to citizenship and to seek public recognition (McGhee, 2004) and in terms of analyses that focus on sexual citizenship as privatised consumption (Evans, 1993). Arguably, one of the effects of discussions of sexual citizenship which focus on the ‘private sphere’ of intimate relations, coupled with the fact that it is ‘the right to privacy’ which has often been the basis for recognition of citizenship claims in relation to sexuality, is a privatisation of sexual citizenship. This reflects a broader shift taking place in the locus of citizenship more generally, what Brown (2006) has described as a *personalisation of citizenship*. This has led to critiques by writers who have problematised the privileging of particular spaces of sexual citizenship through, for example, analyses of sexualities in public spheres (e.g. Bell and Binnie, 2000; Hubbard, 2001) and public sexual citizenship rights (McGhee, 2004). Of course those areas of life that are demarked as ‘personal’ and ‘private’ are closely structured and regulated through public institutions, laws and state policies. This is not the point I want to pursue here; rather I argue for the need to extend such critiques to think about whether this dominant understanding of sexual citizenship translates in geo-political contexts where, for instance, people have little or no access to private spaces and may be compelled to live in the public; or where they are compelled to live in the private and have little or no access to public spaces. In societies where women are expected to observe cultural norms of female seclusion, for example, there are significant social costs associated with going out of the ‘private space’ of the home on their own (Kabeer, 2012).

The focus on the ‘private sphere’ can be interpreted as a narrowing of the field of analysis in other respects by marginalising and obscuring sites where we might think about sexual citizenship, for instance its relation to the economy. A number of contributions to debates on sexual citizenship have addressed the economic basis of sexual citizenship including its relation to privatised consumption (Evans, 1993); to class (Taylor, 2011) and work that has examined the changing political economic structures of contemporary western cities (Bell and Binnie, 2004; Hubbard, 2001). Nevertheless, this remains an underexplored area, as is the relationship between sexual and economic justice (Bedford and Jakobsen, 2009). This reflects the trend in sexuality studies more generally where historically class analysis has been marginal and poverty is an even ‘more neglected focus of critical attention’ (Binnie, 2011: 23). It can also be accounted for in terms of the frameworks of equality that inform recent legislative and policy developments in relation to sexualities equalities. Since the 1990s political claims making in relation to sexual citizenship has been articulated primarily in terms of claims for recognition, rather than in terms of redistributionist politics where poverty is a primary focus as a cause of social exclusion.

Fraser’s (1995, 1998) work on recognition and redistribution and the debates it has sparked, in particular Butler’s (1998) challenge to her argument that sexual injustice is cultural rather than economic, is of particular importance here. Given the available space and focus of this article I do not intend to rehearse and evaluate these debates (see, for example, Hemmings, 2012), but rather underline the lack of attention to economic inequality in the sexual citizenship literature and the implications this has for how sexual citizenship is constituted. For example, that issues of governance in relation to poverty alleviation remain marginal to its analysis might suggest that the concept of sexual citizenship does not have much to offer to debates about ‘larger-scale’ ‘public’ issues of social and economic injustice. One possible effect of this is of querying the relevance of concepts of sexual citizenship in the broader political world: leading to the view that such issues are somehow ‘trivial’ in contrast to more ‘serious’ examinations of social and economic conditions involving food, access to water, shelter, basic needs and so on. In his analysis of sexual citizenship Weeks (1998: 39) alludes to this stating that:

The majority of people on a global scale still have to struggle with getting their daily bread, against the exigencies of extreme poverty, famine, drought, war, authoritarian governments, corruption and violence. Compared to these questions, concerns about sexuality and the body and a sense of self may seem fairly trivial when most people have to struggle just to survive, the worries of the *bien pensant* educated middle class rather than the preoccupations of the embattled majority.

Jolly (2000: 81) makes a similar point in arguing that the relative exclusion of sexuality from development policy and practice agendas suggests ‘the problematic assumption that while in the North people need sex and love, in the South they just need to eat’ (see also Oosterhoff et al., 2014). This, I contest, represents a narrow interpretation of both ‘basic needs’ and ‘sexual citizenship’ and is an argument that can only be sustained if we limit our conceptualisations of sexual citizenship and ignore the links between sexuality, citizenship and poverty, including where sexual citizenship operates as a form of poverty reduction such as, for example, access to citizenship rights through marriage as a form of sustainable livelihood.

## Conclusion

This article has examined the literature on sexuality and citizenship in order to explore how the concept of sexual citizenship has been constituted. In addition to providing a timely and strategic mapping of a diverse literature, it has sought to extend critiques through an analysis of the normative assumptions underpinning notions of sexual citizenship in relation to constructions of the citizen-subject and spaces of sexual citizenship. In seeking to analyse the conceptual limitations to sexual citizenship the key question is not: is the concept of sexual citizenship a distinctly western concept? Clearly, as I have argued, the particularist origins of sexual citizenship have meant that, to date, it has largely been configured in western-centric terms. The more important question is whether and how other constructions of sexual citizenship are imaginable through analyses of struggles over sexual citizenship in different geo-political contexts? The point I am making is not simply that sexual citizenship is not a good enough concept; an inadequate descriptor. It is not a case of add studies of sexualities in the Global South and stir. Rather, as I have tried to argue, any critical reimagining of the concept of sexual citizenship needs to be accompanied by a de-centring of the focus on the Global North if it is to advance theoretical understandings more broadly.

The extension of critiques of sexual citizenship in terms of the framing of the sexual citizen as an atomistic autonomous choosing subject, for example, opens up ways of thinking about how sexual citizenship may operate both in the Global South *and* the North. Bertone (2013), for example, shows how for non-heterosexual youth in Italy sexual citizenship is very much conditioned through the social relations of obligations to family. Similarly, an emphasis on the right to choose may be problematic not only in southern states. In the same way that people who have material, social and cultural capital and resources have an increased ability to make choices and reflect upon themselves and their situations, people constrained by economic resources may be unable to choose to live apart. People living in the UK or the USA, for example, who are constrained by economic realities may be forced to remain living in the same household once their intimate relationship has ended because they cannot afford to move. Similarly, critiques of the centrality of liberal notions of privacy within the sexual citizenship literature prompted by a consideration of sexualities in the Global South afford useful insights into how the privatisation of sexual citizenship can obscure the workings of power associated with forms of citizenship recognition in the North that are typically seen as about privatised ‘choices’ such as, for instance, equal marriage, in addition to marginalising economic dimensions of sexual citizenship.

## Notes

1. Although sexuality entered international human rights discourse only relatively recently, in the early 1990s, there is now a growing body of work concerned with the notion of ‘sexual rights’. It is important to differentiate this literature from that of sexual citizenship which, though there are overlaps, has a different historical configuration and focus.

2. The Global South is a relational concept that emphasises unequal forms of power relations, both historically and contemporaneously, between it and the Global North. Although the article draws on these ‘short-hand terms’, I recognise that such a binary view is overly simplistic.
